# Musculoskeletal pain trajectories of employees working from home during the COVID-19 pandemic

**DOI:** 10.1007/s00420-022-01885-1

**Published:** 2022-06-08

**Authors:** Jodi Oakman, Subas Neupane, Saila Kyrönlahti, Clas-Håkan Nygård, Katrina Lambert

**Affiliations:** 1 Centre for Ergonomics and Human Factors, School of Psychology and Public Health, La Trobe University, Bundoora, 3086 Australia; 2 Unit of Health Sciences, Faculty of Social Sciences, Tampere University, Tampere, Finland; 3 School of Psychology and Public Health, La Trobe University, Bundoora, Australia

**Keywords:** Musculoskeletal pain, Working at home, COVID-19, Trajectory analysis, Quantitative demands, Influence, Workstation

## Abstract

**Objectives:**

In March 2020, the COVID-19 pandemic necessitated a rapid public health response which included mandatory working from home (WFH) for many employees. This study aimed to identify different trajectories of multisite musculoskeletal pain (MSP) amongst employees WFH during the COVID-19 pandemic and examined the influence of work and non-work factors.

**Methods:**

Data from 488 participants (113 males, 372 females and 3 other) involved in the Employees Working from Home (EWFH) study, collected in October 2020, April and November 2021 were analysed. Age was categorised as 18–35 years (*n* = 121), 36–55 years (*n* = 289) and 56 years and over (*n* = 78). Growth Mixture Modelling (GMM) was used to identify latent classes with different growth trajectories of MSP. Age, gender, working hours, domestic living arrangements, workstation comfort and location, and psychosocial working conditions were considered predictors of MSP. Multivariate multinomial logistic regression was used to identify work and non-work variables associated with group membership.

**Results:**

Four trajectories of MSP emerged: high stable (36.5%), mid-decrease (29.7%), low stable (22.3%) and rapid increase (11.5%). Decreased workstation comfort (OR 1.98, CI 1.02, 3.85), quantitative demands (OR 1.68, CI 1.09, 2.58), and influence over work (OR 0.78, CI 0.54, 0.98) was associated with being in the high stable trajectory group compared to low stable. Workstation location (OR 3.86, CI 1.19, 12.52) and quantitative work demands (OR 1.44, CI 1.01, 2.47) was associated with the rapid increase group.

**Conclusions:**

Findings from this study offer insights into considerations for reducing MSP in employees WFH. Key considerations include the need for a dedicated workstation, attention to workstation comfort, quantitative work demands, and ensuring employees have influence over their work.

**Supplementary Information:**

The online version contains supplementary material available at 10.1007/s00420-022-01885-1.

What is already known on this topic?

The significant disruption to traditional working patterns due to the COVID-19 pandemic demonstrated to many organisations that new ways of working are possible. Therefore, it is likely that new models of working arrangements will emerge as the pandemic ends and organisations reassess operational needs; however, evidence is required to understand the requirements for creating sustainable working conditions to prevent the development of musculoskeletal pain.

What this study adds?

Four distinct trajectories of multisite musculoskeletal pain (MSP) were identified in employees working from home during COVID-19 lockdowns. Work and non-work factors were associated with trajectory membership.

How this study might affect research, practice or policy?

Findings support the need for organisations to consider the location and equipment of workstations of employees working at home. in addition, the allocation of quantitative demands and the degree of influence workers have in their roles require consideration.

## Introduction

The disruption to working lives caused by the COVID-19 pandemic is unprecedented. In March 2020, Australia went into the first of multiple lockdowns which required people who could work at home to do so to reduce viral transmission (Douglas et al. 2020). Melbourne, the capital of the southern state of Victoria, experienced strict lockdowns which were in place for 262 days, the longest period across the world. Such a dramatic shift to working conditions has not been previously witnessed and the impact on employees’ physical and mental health is starting to emerge as highly varied. Musculoskeletal pain (MSP) is a significant occupational health burden (Von Bonsdorff et al. 2010; Bevan 2015; Bayattork et al. 2019; Vos et al. 2020; Wu et al. 2020) and associated with reduced work ability and early exit from work. Therefore, understanding the impact of working from home (WFH) on employees’ musculoskeletal pain is an important consideration to inform future strategies designed to reduce the negative health impacts on workers.

A range of factors are typically associated with the complex aetiology of MSP including gender (Collins and O'Sullivan 2015), increasing age (Macpherson et al. 2018), poor psychosocial working conditions (Haukka et al. 2011), and high physical demands (Silva et al. 2016). However, the issue of WFH introduces a range of unique contextual factors which include the location and design of the workstation, impact of domestic living arrangements, and role of managers and supervisors in providing remote leadership and support to their employees. Previous studies have largely focussed on situations where WFH was voluntary and often undertaken as a strategy to improve work life balance, through reduction of commuting, or to provide undistracted working conditions. Mixed impacts on employee health have been reported but in a different context to the current situation (Coggon et al. 2013; Oakman et al. 2020). The significant disruption to traditional working patterns has demonstrated to many organisations that new ways of working are possible. Therefore, it is likely that new models of working arrangements will emerge as the pandemic ends and organisations reassess operational needs. A focus on creating sustainable working conditions will be required to optimise employees physical and mental health.

Musculoskeletal pain in multiple body sites is a common occupational problem and has been linked with more severe consequences compared to single site pain (Nordstoga et al. 2017). Previous studies have explored multisite MSP in a range of occupational groups including health care (Neupane et al. 2016), kitchen workers (Haukka et al. 2012), and municipal workers (Neupane et al. 2018). Although, MSP is a significant problem for white collar employees (Silva et al. 2016), they have received less attention, particularly in the areas of mandatory WFH arrangements. Therefore, the current study aims to examine the developmental trajectories of multisite pain among Australian employees working from home during the COVID-19 pandemic, and to examine the influence of work and non-work factors on multisite MSP over an 18-month period.

## Methods

This study used data collected from the Employees Working from Home (EWFH) study conducted in Australia during the COVID-19 pandemic from October 2020 to November 2021. Sampling and recruitment and a full description of the study profile for the EWFH study have been described elsewhere (Oakman et al. 2022). Briefly, convenience sampling was used to recruit a sample of Australian adults aged 18 or more years who WFH 2 or more days per week during the COVID-19 pandemic. Recruitment occurred via Facebook’s paid service, professional and personal networks, the La Trobe University Facebook page, and LinkedIn.

**Fig. 1:**
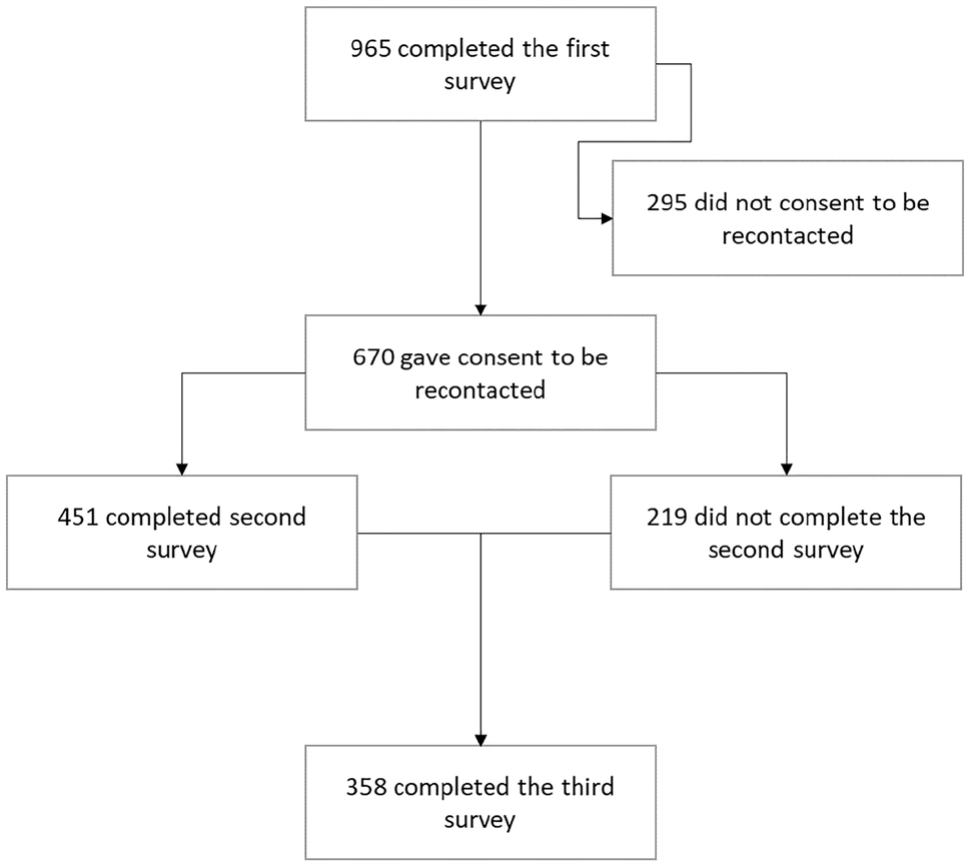
Flow of respondents through the study

Respondents were offered the opportunity to go into a prize draw to win a gift voucher, if they completed the questionnaire and provided contact details. In general, the surveys were open for approximately 4 weeks from the time of opening. Response numbers to the survey at baseline and for the subsequent two time points are outlined in Fig. 1.

### Procedure

Data were collected by questionnaire at three time points via Qualtrics XM software (Qualtrics, Provo, UT). All respondents who consented to be recontacted after the first survey were invited to participate in the second and third surveys. Following the first survey, non-responders were provided with three reminders via email. Responses rates at Survey 2 and 3 were 67% and 53%, respectively. The study flow is outlined in Fig. 1.

Ethics approval was obtained through La Trobe University Human Ethics Research Committee, approval number HEC20388. All study participants were provided with written information about the study. All participants provided informed consent prior to participation.

### Measures

#### Musculoskeletal pain

Musculoskeletal discomfort was recorded separately for five body regions (neck/shoulders, hands/fingers, arms, middle to lower back, and hips/bottom/legs and feet) using a measure with evidence of validity in a number of different industry sectors (Oakman et al. 2014). Question was, “in the past six months have you ever experienced discomfort or pain in part of your body, especially towards the end of your working day or night”. Response options for pain/discomfort frequency ranged from never (1) to almost always (5). For each body region, the score was dichotomised as no pain (0) or having pain (1). The sum score was then ranged from 0 (no pain sites) to 5 (pain in all 5 body regions).

### Other variables

Questions on psychosocial factors were selected from the Copenhagen Psychosocial Questionnaire III (COPSOQ) (Burr et al. 2019). For the current study, constructs (number of items) included: Quantitative Demands (2), Quality of Leadership (2), Vertical Trust (3), Role Clarity (3), and Influence at Work (3). A sample item for quantitative demands was ‘I get behind in my work’. Each item was measured on a 5-point Likert-scale from 1 (Never/hardly ever) to 5 (Always) or 1 (To a very small extent) to 5 (To a very large extent), depending on the item. Mean rating scales for each construct were summed and divided by the number of items. Dimensions were treated as continuous variables in the current analyses, ranging from 1 to 5.

*Work family conflict* (WFC) included five questions from previously validated items (Netemeyer et al. 1996) with a seven-point scale from strongly disagree (1) to strongly agree (7). Average scores across the items were used to construct the final measure as a continuous variable ranging from 1 to 7.

*Job satisfaction* was measured from the item “How pleased are you with your job overall, everything taken into consideration?” with respondents selecting an option from 1 (very unsatisfied) to 5 (very satisfied) (Oakman et al. 2014).

*Demographics* Age was based on the question “What is your age group?” 18–25 years; 26–35 years; 36–45 years; 46–55 years; 56 years and over. The categories were then collapsed to 18–35 years; 36–55 years; 56 years and over. Gender was based on the question “Are you: Male, Female, Other”. Work hours were classified from the following question, “Currently what are your usual working hours (average per week)?”—with those answering ≥ 35 h per week classed as ‘full time’ and others as ‘part-time’.

*Workstation location* Based on the question, developed for this study, “When you are working at home, where do you usually work?”. Three response options were offered: Wherever—“I just find a place somewhere that’s free, such as on the kitchen table or other place”; Separate—“I have my own place in a separate room by myself”; and Interruptions—“I have my own place but in a room that can be busy with other people” (Oakman et al. 2022).

*Workstation comfort* Based on the question *“*How comfortable is your home workstation in comparison to your usual workstation?”, with four response options, very uncomfortable to very comfortable.

*Domestic arrangements* Questions included “Which of the following best describes your usual living arrangements?”, “Do you have caring responsibilities other than children”, and “When you are working at home are children usually at home with you?” A three-level classification was created: Single person household, Adults only, or Dependents.

*Work sector* Based on a question about the sector of employment at the time of the questionnaire.

### Statistical analysis

To describe the course of MSP over the study period, Growth Mixture Modelling (GMM) analyses were used to identify latent classes with different growth trajectories of number of reported pain sites over the three time points. These models are less restrictive than a latent class analysis, as the GMM accounts for between-subject heterogeneity within the latent classes by including random effects. Respondents were required to have at least two survey responses to be included in the trajectory modelling. GMM models with one to five classes were examined, with each model being run 50 times with different starting values to ensure the optimal solution was found instead of local maxima. The optimal solutions for each class number were compared and the Bayesian information criterion (BIC) was used to select the best fit model (see Fig. 1; Table S1). Trajectory analyses were run with the ‘*hlme*’ function from the R package ‘*lcmm*’ (Proust-Lima et al. 2016).

Individuals were matched to a latent class using posterior probabilities, with each individual allocated to the group for which the probability was the highest (Berlin et al. 2014). Demographic differences between participants in each group were calculated using the chi-squared test of independence. Due to small numbers, the *n* = 3 respondents who identified their gender as ‘Other’ were excluded from further exploratory analysis. A multinomial logistic regression model was used to determine the associations between predictors at baseline and group membership based on the GMM. Multinomial regression analysis was used, because the response variable has several unordered categories. Odds ratios (OR) with 95% confidence intervals (CI) were determined, comparing membership in each group to the chosen reference category which was low stable.

All statistical analysis was performed in R version 4.1.1 “Kick Things” (R Core Team 2021). All tests of statistical significance were two-tailed, and *p* < 0.05 was considered significant.

## Results

**Fig. 2:**
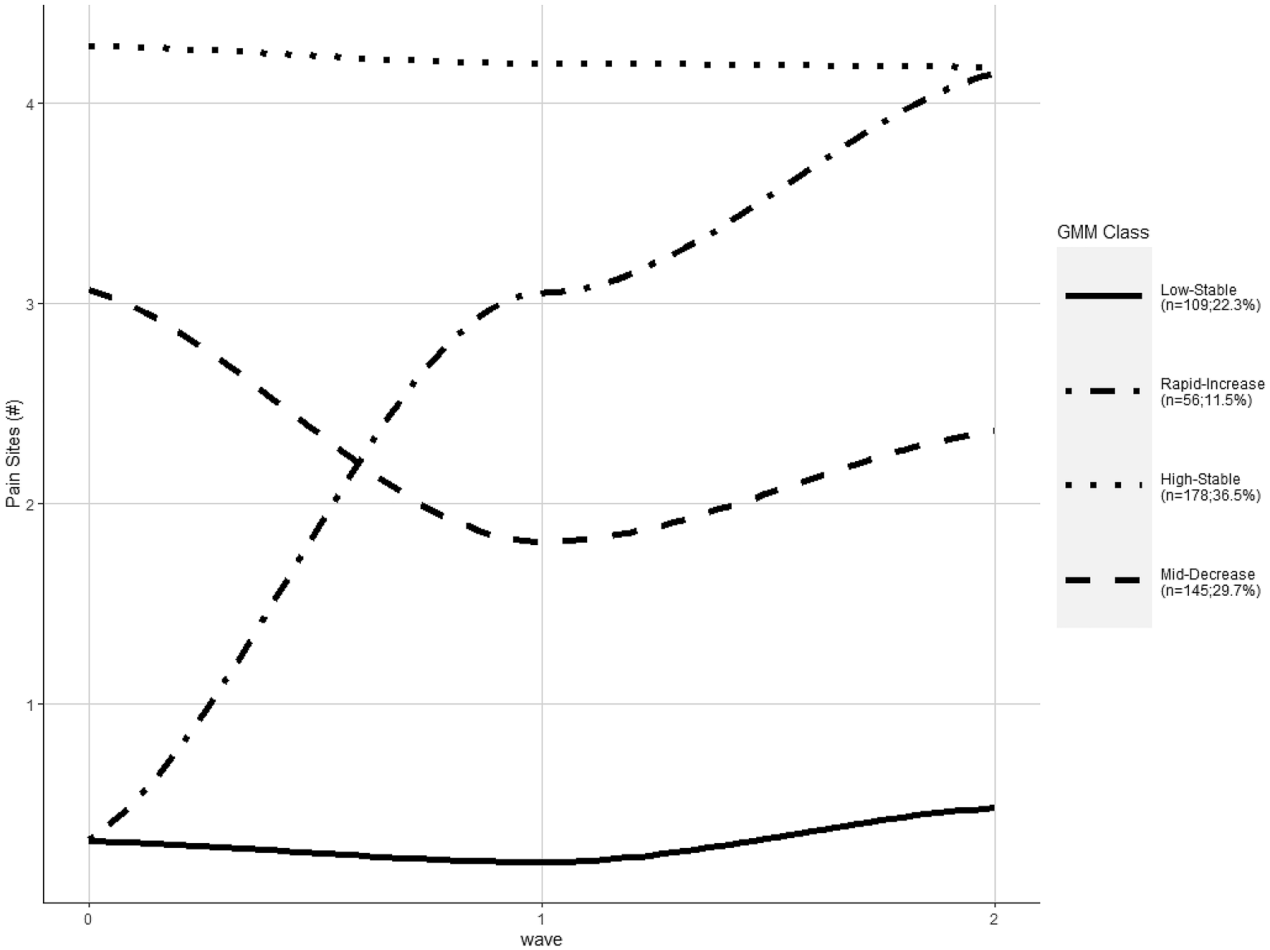
Profiles of MSP during WFH during the COVID-19 pandemic, data collected at three time points: October 2020, April and November 2021

Three surveys were completed by 303 respondents with a further 185 respondents completing two of the surveys—first and second or first and third—for a total sample of *n* = 488. The four-class solution was selected as the best fit for the trajectory modelling (Fig. 2). The largest group high stable (36.5% of respondents) was characterised by a high number of pain sites which remained constant throughout the study. The second largest group, mid-Decreasing (29.7% of respondents) was characterised by the number of pain sites dipping during the first follow-up compared to baseline. The low-stable group accounted for 22.3% of the respondents and was characterized by a low number of pain sites reported with minimal changes throughout the study. The rapid-increase group was small (11.5% of respondents) with participants in this group reporting a low number of pain sites at baseline that subsequently increased during follow-up.

The four trajectory groups showed demographic differences in age, gender and state of residence (Table 1). The optimal low-stable group were older, include more men and living in states other than Victoria (which had experienced the longest lockdown periods). Groups were unrelated to baseline domestic arrangements and number of hours worked but were associated with the level of workstation comfort compared to their pre-pandemic office location. The members of the high stable and mid-decrease groups reported a higher number of pain sites at baseline. Further details on domestic arrangements are provided in Table S2.

**Table 1:** Demographic differences between the four pain site trajectory groups

		Low-stable (*N* = 109)		Rapid-increase (*N* = 56)		High-stable (*N* = 178)		Mid-decrease (*N* = 145)		p*
*Age*										0.007
18–35 years		16 (14.68%)		15 (26.79%)		46 (25.84%)		44 (30.34%)		
36–55 years		72 (66.06%)		35 (62.50%)		99 (55.62%)		83 (57.24%)		
56 years and over		21 (19.27%)		6 (10.71%)		33 (18.54%)		18 (12.41%)		
*Gender*										0.013^†^
Male		37 (33.94%)		15 (26.79%)		32 (17.98%)		29 (20.00%)		
Female		72 (66.06%)		41 (73.21%)		145 (81.46%)		114 (78.62%)		
Other		0 (0%)		0 (0%)		1 (0.56%)		2 (1.38%)		
*State*										0.003
Victoria		84 (77.06%)		47 (83.93%)		165 (92.70%)		125 (86.21%)		
Other		25 (22.94%)		9 (16.07%)		13 (7.30%)		20 (13.79%)		
*Domestic arrangements*										0.831
Single person household		11 (10.09%)		7 (12.50%)		19 (10.67%)		21 (14.48%)		
Adults only		50 (45.87%)		28 (50.00%)		80 (44.94%)		59 (40.69%)		
Dependents		48 (44.04%)		21 (37.50%)		79 (44.38%)		65 (44.83%)		
*Employment sector*										
Education and training		34 (31.19%)		17 (30.36%)		71 (39.89%)		54 (37.24%)		na
Financial and insurance services		3 (2.75%)		2 (3.57%)		3 (1.69%)		3 (2.07%)		
Healthcare and social assistance		19 (17.43%)		11 (19.64%)		27 (15.17%)		18 (12.41%)		
Media and telecommunication		3 (2.75%)		4 (7.14%)		6 (3.37%)		6 (4.14%)		
Professional services		19 (17.43%)		8 (14.29%)		42 (23.60%)		40 (27.59%)		
Public administration and safety		19 (17.43%)		8 (14.29%)		15 (8.43%)		10 (6.90%)		
Transport, postal and warehousing		2 (1.83%)		0 (0.00%)		1 (0.56%)		5 (3.45%)		
*Average hours worked*										0.385^‡^
Full time		80 (73.39%)		44 (78.57%)		136 (76.40%)		100 (68.97%)		
26–34 h		16 (14.68%)		4 (7.14%)		20 (11.24%)		26 (17.93%)		
21–25 h		8 (7.34%)		5 (8.93%)		16 (8.99%)		12 (8.28%)		
15–20 h		2 (1.83%)		3 (5.36%)		5 (2.81%)		6 (4.14%)		
14 h or less		3 (2.75%)		0 (0.00%)		1 (0.56%)		1 (0.69%)		
*Workstation location*										0.133
Work wherever		7 (6.42%)		11 (19.64%)		21 (11.80%)		22 (15.17%)		
Separate room		77 (70.64%)		32 (57.14%)		105 (58.99%)		84 (57.93%)		
Interruptions		25 (22.94%)		13 (23.21%)		52 (29.21%)		39 (26.90%)		
*Workstation comfort*										
Less comfortable		37 (33.94%)		26 (46.43%)		109 (61.24%)		88 (60.69%)		< 0.001
No change		48 (44.04%)		16 (28.57%)		47 (26.40%)		33 (22.76%)		
More comfortable		24 (22.02%)		14 (25.0%)		22 (12.36%)		24 (16.55%)		
*Psychosocial conditions*										
Work–family conflict		3.14 ± 1.56		3.31 ± 1.72		3.95 ± 1.71		3.86 ± 1.58		< 0.001
Job satisfaction		3.98 ± 1.03		3.96 ± 0.89		3.80 ± 0.96		3.79 ± 1.00		0.144
Quantitative demands		2.23 ± 0.86		2.44 ± 0.90		2.68 ± 0.82		2.52 ± 0.78		< 0.001
Quality of leadership		3.65 ± 1.20		3.58 ± 1.18		3.37 ± 1.11		3.44 ± 1.19		0.178
Vertical trust		3.83 ± 1.06		3.65 ± 0.97		3.50 ± 1.07		3.64 ± 1.00		0.041
Role clarity		3.98 ± 0.82		3.83 ± 0.79		3.69 ± 0.83		3.71 ± 0.89		0.012
Influence at work		3.40 ± 0.95		3.34 ± 0.76		3.00 ± 0.94		3.17 ± 0.87		0.002
*Baseline pain sites*										
No pain		89 (81.7%)		45 (80.4%)		0 (0%)		0 (0%)		
Neck		17 (15.6%)		10 (17.9%)		176 (98.9%)		138 (95.2%)		
Hand/wrist		2 (1.8%)		0 (0%)		139 (72.5%)		57 (39.3%)		
Shoulder/arm		1 (0.9%)		0 (0%)		120 (67.4%)		38 (26.2%		
Back		12 (11.0%)		5 (8.9%)		173 (97.2%)		130 (89.7%)		
Feet		9 (8.3%)		2 (3.6%)		157 (88.2%)		100 (69.0%)		

*p value of the Pearson's Chi-squared test//Kruskal–Wallis rank sum test

^†^Category ‘Other’ excluded, comparing male to female

^‡^Comparison between ‘Full time’ and ‘Not full time’ (being all other categories)

Increasing quantitative demands significantly increased the odds of being in a group other than low-stable (Table 2). Being female predicted membership in the high stable (OR 2.81 95% CI 1.43, 5.55) and mid-decrease (OR 1.99 95% CI 1.01, 3.91) groups, as did a decreased workstation comfort (OR 1.98 95% CI 1.02, 3.85 and OR 2.31 95% CI 1.15, 4.66, respectively). Each increase of one in the scale influence at work was associated with a decrease in odds of belonging to the high stable group (OR 0.78 95% CI 0.54, 0.98). The mid-decrease had higher odds of work family conflict (OR 1.25 95% CI 1.00, 1.58). Being required to work wherever there was free space, such as on the kitchen table rather than having a separate room, significantly increased the odds of being in the rapid-increase group (OR 3.86 95% CI 1.19, 12.52).

**Table 2:** Multivariate multinomial logistic regression associations between pain site trajectories and baseline predictors

	Rapid-increase v low-stable OR (95%CI)	High-stable v low-stable OR (95%CI)	Mid-decrease v low-stable OR (95%CI)
*Age*			
18–35 years	Ref	Ref	Ref
36–55 years	0.58 (0.22, 1.52)	0.53 (0.24, 1.18)	0.46 (0.21, 1.03)
56 years and over	0.44 (0.12, 1.61)	0.92 (0.34, 2.47)	0.45 (0.16, 1.28)
*Gender*			
Male	Ref	Ref	Ref
Female	1.22 (0.53, 2.81)	2.81 (1.43, 5.55)**	1.99 (1.01, 3.91)**
*Average hours worked*			
< 25 h	Ref	Ref	Ref
26–34 h	0.69 (0.13, 3.74)	0.80 (0.23, 2.74)	1.46 (0.43, 4.98)
35+ hrs	1.04 (0.28, 3.83)	0.88 (0.32, 2.39)	0.88 (0.31, 2.46)
*Workstation location*			
Separate room	Ref	Ref	Ref
Interruptions	1.01 (0.40, 2.56)	1.28 (0.65, 2.52)	1.03 (0.51, 2.08)
Work wherever	3.86 (1.19, 12.52)*	1.21 (0.42, 3.54)	1.44 (0.50, 4.19)
*Domestic arrangements*			
Single person household	Ref	Ref	Ref
Adults only	0.93 (0.27, 3.17)	0.73 (0.27, 2.01)	0.42 (0.16, 1.13)
Dependents	0.65 (0.17, 2.42)	0.82 (0.29, 2.35)	0.47 (0.17, 1.32)
*Workstation comfort*			
Stayed the same	Ref	Ref	Ref
Decreased	1.84 (0.76, 4.45)	1.98 (1.02, 3.85)*	2.31 (1.15, 4.66)*
Increased	1.70 (0.62, 4.68)	0.78 (0.33, 1.83)	1.37 (0.59, 3.19)
*Psychosocial conditions*			
Work–family conflict	0.96 (0.72, 1.29)	1.12 (0.89, 1.40)	1.25 (1.00, 1.58)
Job satisfaction	1.27 (0.81, 2.01)	1.27 (0.91, 1.78)	1.04 (0.74, 1.47)
Quantitative demands	1.44 (1.01, 2.47)*	1.68 (1.09, 2.58)**	1.11 (0.72, 1.71)
Quality of leadership	0.98 (0.66, 1.45)	0.95 (0.70, 1.30)	0.92 (0.67, 1.25)
Vertical trust	0.79 (0.48, 1.30)	0.95 (0.64, 1.41)	1.06 (0.71, 1.59)
Role clarity	1.15 (0.63, 2.08)	0.90 (0.57, 1.42)	0.92 (0.58, 1.47)
Influence	1.22 (0.76, 1.96)	0.78 (0.54, 0.98)*	1.06 (0.73, 1.55)

*p < 0.05 **p < 0.01 ***p < 0.001

## Discussion

In this study of employees working from home during the COVID-19 pandemic, we found four distinct trajectories of multisite MSP across the three rounds of follow-up: high stable, mid-decrease, low stable and rapid increase. Approximately one-third of employees belonged to the high stable group and about one in ten to the rapid increase in MSP group. Based on the modelling, quantitative demands were a common predictor for membership of the high stable and the rapid increase group. Work location was relevant for the rapid increasing group, whilst workstation comfort and the degree of influence were associated with membership of the high stable trajectory. We were unable to locate previous studies which examined the predictors of MSP over time whilst in mandatory WFH conditions, thus these findings offer unique insights into potential strategies for organisations to implement for employees who may continue to WFH beyond the current COVID-19 pandemic.

The distinct differences between the four MSP trajectories support the importance of identifying relevant predictive factors, which can then be appropriately targeted as part of a comprehensive occupational health prevention strategy. The rapid increasing group, although the smallest, offers insights for occupational health professionals and highlights the importance of having an adequate workspace whilst working at home. During the peak of the pandemic when lockdowns where in situ and schools were closed, this was very challenging for many employees and necessitated working from wherever they could find. However, beyond the pandemic, when working from home is part of a negotiated employment pattern, the importance of a dedicated space may require negotiation with workers about what will constitute an appropriate workstation to support sustainable WFH. Prior to the pandemic, many organisations required workstation assessments as part of WFH agreements; however, the rapid shift to WFH did not enable time and resources to facilitate remote workstation assessments for all employees. Beyond the pandemic, hybrid work models will require negotiation about who is responsible for equipment provision, in both “places of work”.

Quantitative demands, including workload distribution, time pressures and quantity, were important predictors of the high stable and rapid increasing groups, which comprised nearly 50% of the employees. An emerging issue from the pandemic relates to the challenges of managing workload and the close relationships with boundary setting, that is the division between home and work (Allen et al. 2021). Multiple competing demands were experienced by workers trying to juggle work and home life. For managers and supervisors, the COVID-19 pandemic and WFH resulted in new challenges in the provision of effective leadership remotely. Without any training, managers previously accustomed to leading mostly collocated teams, had to adapt and modify how they interacted and the ways in which they provided support to their direct reports. For employees, job roles needed to adapt to the conditions enforced by WFH, whilst some job tasks were easily shifted others were more difficult and required modification to suit new working arrangements (Wang et al. 2021). The role for managers and supervisors in setting realistic expectations about workloads is highlighted in the current study, where quantitative demands emerge as an important predictor of MSP. Although the relationship between quantitative demands and MSP has previously been identified in computer users, particularly females, these were not in the WFH situation (Larsman et al. 2006; Johnston et al. 2009). The concept of quantitative demands has been raised as challenging by a number of researchers who suggest that it is poorly defined and therefore hard to address (Kristensen et al. 2004). For the purpose of workplace prevention, clear communication about the inherent requirements of work, and setting deadlines collaboratively which enable employees to have some control over their workload may offer reasonable practical solutions to a complex problem, consistent with good job design principles (Wang et al. 2021).

Employees reporting having a low degree of influence was associated with membership of the high stable group. Research prior to the COVID-19 pandemic has found influence or job control as an important mechanism which enables employees to manage their MSP, through modifying their work tasks, hours of work, and when they schedule breaks. A large review by Lang et al. (2012) found low job control significantly increased the risk of developing musculoskeletal symptoms, with OR 1.30 (95% CI 1.11–1.52). A large Belgian study found that higher levels of job control were associated with reduced risk of longer term sickness absence due to MSP in middle aged workers (Janssens et al. 2014). However, this pre-pandemic research was not undertaken in the WFH context so whilst it supports the importance of influence, the current study extends these findings to a new location and supports the need to provide workers with opportunities for influence in their work.

Work–family conflict arose as important for those in the mid-decrease group, along with being female and having low workstation comfort. Prior to the pandemic, WFH was considered a benefit offered by organisations which enabled better integration of work and family, and a strategy to reduce WFC (Felstead and Henseke 2017; Oakman et al. 2020). However, the pressures of the pandemic and mandated WFH removed employee control, with mixed impacts (Collins et al. 2021). Whilst some positive benefits from WFH during the pandemic have been reported (Moens et al. 2021), negative gendered impacts are also emerging, with females experiencing higher levels of WFC compared to males (Carli 2020). The longer term impacts of WFC arising from WFH will require careful scrutiny, as organisations adapt to new models of working, to ensure that any negative impacts of hybrid work patterns do not have unintended and gendered consequences. The relationship between WFC and MSP has been previously reported as significant (Weale et al. 2018, 2021) but in more traditional work settings where WFH was optional rather than mandated.

Traditionally, prevention of MSP has been focussed on physical aspects of work and for office work the workstation set up and equipment (Macdonald and Oakman 2015). The results from this study support that whilst workstation comfort is an important factor, other work factors are also important influences on the development of MSP, such as the demands, influence, and work family conflict. Good ergonomic practice should take into account the systems of work related to an individual (Wilson 2014), but often, the focus is reduced to addressing the physical workstation set up, particularly in a WFH situation. The current study provides support for the need to take a more nuanced approach to MSP prevention, not only during the COVID-19 pandemic but beyond to address the changing nature of the work environment that is likely to become the “new normal”.

### Strengths and limitations

A strength of the study was the prospective design with the three data collection waves over an 18-month period during the COVID-19 pandemic, which impacted global working conditions. Survey questions used previously validated measures with only a few exceptions (Neupane et al. 2017). In relation to MSP, specificity of body sites was not particular to this study, a body chart was used to assist participants in locating the regions of their MSP and may have aided the accuracy of responses. Data were not collected on participants MSP levels prior to the COVID-19 pandemic. The question regarding workstation location was developed for the purpose of the current study as no suitable published question was identified.

The EWFH study population is a convenience sample, based in Australia, which may restrict the generalisability of the results. The sample contains a higher proportion of women compared to men. Whilst the sample size is adequate (de Jong et al. 2019), the relatively small size of some groups does impact the precision of the risk estimates. The drop out of participants across the study is a further limitation. All measures are based on self-report, objective measures were not practicable during the pandemic situation in which this data were collected. Finally, the data for the present study were collected during the COVID-19 pandemic; as such the broader environmental context involved significant changes to many aspects of lives beyond work and is an important factor to consider in the interpretation of the study results.

## Concluding remarks

This longitudinal study of employees WFH during the COVID-19 pandemic adds to the current limited evidence on the impact of mandatory WFH on MSP over time. The results provide insights into future policy considerations for employers who wish to optimise working conditions for their employees working remotely beyond the pandemic. Further studies over time are suggested to examine the longer term impacts, both positive and negative, of WFH.

## Supplementary Information

Below is the link to the electronic supplementary material.

Supplementary file1 (DOCX 555 KB)

## Data Availability

The datasets generated during and/or analysed during the current study are available from the corresponding author on reasonable request.
