# Supplementary material for: Musculoskeletal pain trajectories of employees working from home during the COVID-19 pandemic
Source: Int Arch Occup Environ Health. 2022 Jun 8;95(9):1891–901. doi: 10.1007/s00420-022-01885-1 (PMC9175522; doi:10.1007/s00420-022-01885-1)
Supplement: Supplementary file 1 — Supplementary file1 (DOCX 555 KB) [file 420_2022_1885_MOESM1_ESM.docx]

**Supplementary Table 1**

Table S1: Model fit indices for the Growth Mixture Modelling to determine trajectories of number of pain sites.

| Number of classes | Maximum  log-likelihood | Bayesian Information Criterion (BIC) | Sample Adjusted BIC | Entropy | Class proportions % \| Mean Posterior Probabilities | | | | |
| --- | --- | --- | --- | --- | --- | --- | --- | --- | --- |
|  |  |  |  |  | 1 | 2 | 3 | 4 | 5 |
| 1 | -3094.916 | 6216.518 | 6203.879 | 1.000 | 100.00\| - |  |  |  |  |
| 2 | -3047.114 | 6141.037 | 6118.808 | 0.702 | 66.95\|0.9170 | 33.04\|0.9094 |  |  |  |
| 3 | -3030.570 | 6128.012 | 6096.256 | 0.594 | 36.90\|0.7135 | 35.54\|0.8334 | 27.56\|0.9037 |  |  |
| 4 | -2942.795 | 5992.584 | 5941.775 | 0.694 | 22.34\|0.7487 | 11.48\|0.8283 | 36.48\|0.8045 | 29.71\|0.8399 |  |
| 5 | -2975.904 | 6038.741 | 5997.458 | 0.672 | 33.42\|0.8352 | 24.82\|0.7404 | 30.42\|0.8290 | 6.23\|0.8601 | 5.11\|0.8486 |

**Table S2: Detail of domestic arrangements.**

|  | Low-Stable  (N=109) | Rapid-Increase  (N = 56) | High-Stable  (N=178) | Mid-Decrease  (N=145) |
| --- | --- | --- | --- | --- |
| Living Arrangement |  |  |  |  |
| Alone | 11 (10.09%) | 7 (12.50%) | 21 (11.80%) | 22 (15.17%) |
| With adults and no children | 58 (53.21%) | 29 (51.79%) | 100 (56.18%) | 68 (46.90%) |
| With children and adults | 38 (34.86%) | 19 (33.93%) | 51 (28.65%) | 51 (35.17%) |
| With children and no adults | 2 (1.83%) | 1 (1.79%) | 6 (3.37%) | 4 (2.76%) |
| Number of Children |  |  |  |  |
| None | 69 (63.30%) | 36 (64.29%) | 121 (67.98%) | 90 (62.07%) |
| 1 | 11 (10.09%) | 6 (10.71%) | 15 (8.43%) | 17 (11.72%) |
| 2 | 24 (22.02%) | 11 (19.64%) | 31 (17.42%) | 34 (23.45%) |
| 3+ | 5 (4.59%) | 3 (5.36%) | 11 (6.18%) | 4 (2.76%) |
| Age Groups* |  |  |  |  |
| Baby | 1 | 2 | 0 | 7 |
| Pre-school | 5 | 6 | 8 | 13 |
| Primary School | 23 | 13 | 36 | 36 |
| Secondary School | 23 | 5 | 45 | 14 |
| Caring Responsibilities other than children |  |  |  |  |
| No | 94 (86.24%) | 50 (89.29%) | 138 (77.53%) | 127 (87.59%) |
| Yes | 15 (13.76%) | 6 (10.71%) | 40 (22.47%) | 18 (12.41%) |
| **Domestic Arrangements** |  |  |  |  |
| Single person household | 11 (10.09%) | 7 (12.50%) | 19 (10.67%) | 21 (14.48%) |
| Adults only | 50 (45.87%) | 28 (50.00%) | 80 (44.94%) | 59 (40.69%) |
| Dependents | 48 (44.04%) | 21 (37.50%) | 79 (44.38%) | 35 (44.83%) |
